# Brexpiprazole Reduces 5-HT7 Receptor Function on Astroglial Transmission Systems

**DOI:** 10.3390/ijms23126571

**Published:** 2022-06-12

**Authors:** Kouji Fukuyama, Eishi Motomura, Motohiro Okada

**Affiliations:** Department of Neuropsychiatry, Division of Neuroscience, Graduate School of Medicine, Mie University, Tsu 514-8507, Japan; k-fukuyama@clin.medic.mie-u.ac.jp (K.F.); motomura@clin.medic.mie-u.ac.jp (E.M.)

**Keywords:** brexpiprazole, astrocyte, metabolic syndrome, antipsychotics

## Abstract

Several atypical antipsychotics exert mood-stabilising effects via the modulation of various monoamine receptors and intracellular signallings. Recent pharmacodynamic studies suggested that tripartite synaptic transmission can contribute to the pathophysiology of schizophrenia and mood disorders, their associated cognitive impairment, and several adverse reactions to atypical antipsychotics. Therefore, to explore the mechanisms underlying the antidepressive mood-stabilising and antipsychotic effects of brexpiprazole (Brex), we determined the effects of subchronic administration of therapeutically relevant concentrations/doses of Brex on the protein expression of 5-HT receptors, connexin43, cAMP levels, and intracellular signalling in cultured astrocytes and rat hypothalamus using ultra-high-pressure liquid chromatography with mass spectrometry and capillary immunoblotting systems. Subchronic administration of a therapeutically relevant concentration of Brex (300 nM) downregulated both 5-HT1A (5-HT1AR) and 5-HT7 (5-HT7R) receptors, in addition to phosphorylated Erk (pErk), without affecting phosphorylated Akt in the astroglial plasma membrane. Subchronic administration of 300 nM Brex decreased and increased phosphorylated AMPK and connexin43, respectively, in the astroglial cytosol fraction. A therapeutically relevant concentration of Brex acutely decreased the astroglial cAMP level, whereas, under the inhibition of 5-HT1AR, Brex did not affect astroglial cAMP levels. However, the 5-HT7R-agonist-induced increased astroglial cAMP level was inhibited by Brex. In contrast to the in vitro study, systemic subchronic administration of effective doses of Brex (3 and 10 mg/kg/day for 14 days) increased the cAMP level but did not affect phosphorylated AMPK in the rat hypothalamus. These results suggest several complicated pharmacological features of Brex. Partial 5-HT1AR agonistic action predominates in the low range of therapeutically relevant concentrations of Brex, whereas in the high range, 5-HT7R inverse agonist-like action is overlapped on the 5-HT1A agonistic action. These unique suppressive effects of Brex on 5-HT7R play important roles in the clinical features of Brex regarding its antidepressive mood-stabilising actions.

## 1. Introduction

**Figure 1:**
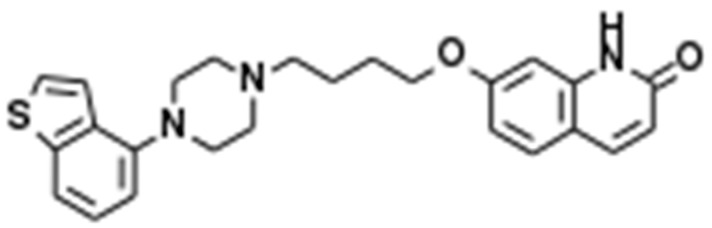
Chemical structure of brexpiprazole (Brex), 7-[4-[4-(2,3-Dichlorophenyl)-1-piperazinyl]butoxy]-3,4-dihydro-2(1H)-quinolinone.

A novel mood-stabilising atypical antipsychotic agent, brexpiprazole (Brex), 7-[4-[4-(2,3-Dichlorophenyl)-1-piperazinyl]butoxy]-3,4-dihydro-2(1H)-quinolinone (Figure 1), has been approved for the treatment of schizophrenia and major depression in several countries, including the USA, EU, Canada, Australia, and Japan [1]. Several meta-analyses and systematic reviews confirmed that the antipsychotic effects of Brex for the treatment of patients with schizophrenia in the acute phase and for relapse prevention are comparable to those of other atypical antipsychotics [2,3]. A recent meta-analysis study provided findings for Brex as an efficacious augmentation agent in antidepressant-resistant major depression, comparable to other mood-stabilising atypical antipsychotics [4]. Indeed, Brex was approved as an adjunctive treatment to oral antidepressants in major depression based on three clinical trials that showed relatively rapid improvement (within six weeks) of depressive symptoms [5,6,7]. In addition to these clinical advantages, Brex has also been considered the safest option in patients with a risk of developing metabolic complications since it is listed among the best atypical antipsychotics associated with metabolic outcomes [8].

The major mechanisms of Brex are considered to be similar to those of aripiprazole: partial agonism with high affinity at the dopamine D2 receptor (D2R) (Ki  =  0.30 nM) and 5-HT1A receptor (5-HT1AR; Ki = 0.12 nM) (Table 1) [9,10]. Furthermore, the intrinsic activities at D2R and 5-HT1AR are lower than those for aripiprazole [11]. However, the clinical features of Brex cannot be fully explained by its partial agonisms to D2R and 5-HT1AR alone [12]. A pharmacogenomic study revealed that 5-HT7 receptor (5-HT7R) antagonism contributes to the pathophysiology of mood disorders and schizophrenia [13]. Additionally, functional abnormalities of tripartite synaptic transmission play important roles in the pathophysiology of several psychiatric disorders [13,14,15]. Indeed, the expression of several monoamine receptors, such as 5-HT1AR, D2R, 5-HT7R, and 5-HT2A receptor (5-HT2AR), in astrocytes has been identified [12,16,17,18]. These preclinical findings suggest that the involvement of astroglial monoaminergic transmission provides an opportunity to expand a pathophysiological hypothesis of neuropsychiatric disorders, such as novel monoaminergic tripartite synaptic transmission, including astroglial transmission [13,19].

According to the monoaminergic tripartite synaptic transmission hypothesis, it has been suggested that the pharmacodynamic profile associated with astroglial transmission associated with hemichannels correlates with efficacy in mood disorders [15,19]. Connexin43-containing astroglial hemichannels in the plasma membrane fraction of cultured astrocytes were found to be upregulated by several mood-stabilising atypical antipsychotics—clozapine, quetiapine, and zotepine [12,28,29]—but downregulated by the antidepressant vortioxetine [18]. Interestingly, a selective 5-HT-transporter-inhibiting antidepressant, escitalopram, did not affect connexin43 protein expression in the astroglial plasma membrane under the 5-HT-free condition [18]. Contrary to the results of in vitro study, systemic administration of fluoxetine and duloxetine increased the expression of connexin43 mRNA or protein in the total lysate [30,31], whereas lipopolysaccharide- and corticosterone-induced upregulation of connexin43 was suppressed by fluoxetine [32,33]. These previous findings suggest that the activation and suppression of tripartite synaptic transmission associated with astroglial hemichannels are involved in mood-stabilising and antidepressive actions, respectively [15]. Indeed, a mood-stabilising antipsychotic, lurasidone, exhibits exceptional clinical and preclinical characteristics among the mood-stabilising antipsychotics since lurasidone is more effective in its antidepressive action than its antimanic action, possibly due to its inhibitory effects on astroglial L-glutamate release through hemichannels via its 5-HT7R inverse agonistic action [34]. Additionally, the 5-HT7R inverse agonistic action of lurasidone plays an important role in the mechanisms of a lower risk of developing metabolic complications via the suppression of adenosine monophosphate-activated protein kinase (AMPK) signalling [34]. 

Considering the similar clinical features of lurasidone and Brex, an antidepressive-dominant action, and a lower risk of developing metabolic complications, the binding affinity of Brex to 5-HT7R (Ki = 3.7 nM) can provide us with information regarding the scientific effectiveness in a pharmacodynamic examination of the effects of Brex on astroglial transmission associated with 5-HT7R [9,10]. Although 5-HT7R-binding atypical antipsychotics have been reported, and many of these agents have shown 5-HT7R inhibition, it has not been clarified whether they are antagonists or inverse agonists [13]. Our recent study demonstrated that Brex weakly enhanced astroglial L-glutamate release through activated astroglial hemichannels, whereas the mechanisms of Brex were not identical to those of clozapine or quetiapine [12]. Clozapine, quetiapine, and zotepine enhanced the trafficking of connexin43 to the plasma membrane via the activation of protein kinase B (Akt) signalling, but the action of Brex was not dependent on Akt signalling [12], similar to that of lurasidone (high-affinity 5-HT7R inverse agonist) [34]. These previous findings suggest that the unclarified effects of Brex on 5-HT7R possibly contribute to the action of Brex on tripartite synaptic transmission associated with astroglial hemichannels. Therefore, the detailed mechanisms of Brex on 5-HT7R and connexin43 remain to be clarified. Based on these previous findings, we aimed to explore the effects of Brex on tripartite synaptic transmission associated with 5-HT7R, determined in the present study via subchronic administration of Brex on astroglial transmission using a capillary immunoblotting system. Additionally, when Brex inhibits AMPK signalling or 5-HT7R, the subsequent effects of subchronic systemic administration of Brex on AMPK signalling in the hypothalamus possibly contribute to the mechanisms of a lower risk of metabolic complications [34].

## 2. Results

### 2.1. Effects of Brex on Astroglial Signalling

To clarify the mechanisms underlying the time-dependent effects of Brex on several astroglial proteins associated with 5-HT signalling, the effects of subchronic administration of a therapeutically relevant concentration of Brex on the expression of proteins such as 5-HT1AR, 5-HT7R, phosphorylated extracellular signal-regulated kinase (pErk), phosphorylated protein kinase B (pAkt), and phosphorylated adenosine monophosphate-activated protein kinase (pAMPK) in the plasma membrane or cytosol fractions of astrocytes were determined using a capillary immunoblotting system. The therapeutically relevant serum concentrations of Brex are reported to range from 90 nM to 300 nM [35,36]. In accordance with a previous clinical report, in the present study, the cortical primary cultured astrocytes were subchronically (for 7 and 14 days) administered 300 nM Brex [12]. The major procedures of preparation of the cultured astrocytes and the study design are described in the following Section 4.2. according to our previous studies [12,18,34,37,38,39,40,41,42,43].

#### 2.1.1. Effects of Subchronic Administration of a Therapeutically Relevant Concentration of Brex on the Protein Expression of 5-HT1A and 5-HT7R in the Plasma Membrane Fraction of Astrocytes

**Figure 2:**
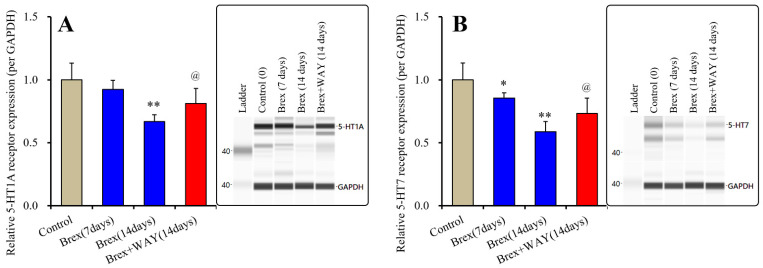
Effects of subchronic administration (7 and 14 days) of therapeutic relevant concentration of Brex (Brex: 300 nM) and interaction between Brex and 5-HT1A receptor (5-HT1AR) antagonist WAY100635 (WAY: 10 μM) on protein expression of 5-HT1A (panel **A**) and 5-HT7 (panel **B**) receptor in the plasma membrane fraction of cortical primary cultured astrocytes. In left side histograms, ordinate: mean ± SD (*n* = 6) of the relative protein level of 5-HT1AR and 5-HT7R per GAPDH. * *p* < 0.05, ** *p* < 0.01: relative to control (Brex-free) by one-way analysis of variance (ANOVA) with Tukey’s post-hoc test, and @ *p* < 0.05: relative to Brex for 14 days by Student’s *T*-test. Right side panels indicate their pseudo-gel images using capillary immunoblotting.

Subchronic administration of a therapeutically relevant concentration of Brex (300 nM) for 14 days decreased 5-HT1AR expression, whereas administration for 7 days did not affect its expression (F(2,15) = 21.8(*p* < 0.01)); however, subchronic administration of a therapeutically relevant concentration of Brex (300 nM) for 7 or 14 days decreased 5-HT7R expression in the plasma membrane fractions (F(2,15) = 20.4(*p* < 0.01)) (Figure 2).

To clarify the mechanisms of time-dependent downregulation of 5-HT1AR and 5-HT7R induced by Brex, the cultured astrocytes were also subchronically administered 300 nM Brex along with the 5-HT1AR antagonist WAY100635 (10 μM) for 14 days. In accordance with our expectations, WAY100635 prevented the downregulation of 5-HT1A and 5-HT7R induced by subchronic administration of Brex (Figure 2).

The therapeutically relevant concentration of Brex also downregulated both 5-HT1AR and 5-HT7R, but the downregulation of 5-HT7R induced by Brex had a faster onset than did that of 5-HT1AR. The subchronic administration of 5-HT1AR agonist and 5-HT7R inverse agonist downregulated both 5-HT1A and 5-HT7R; however, the downregulation of 5-HT1A and 5-HT7R induced by 5-HT7R inverse agonist is faster in its onset that that induced by 5-HT1AR agonist [18,34]. Taken together with the previous findings, these results suggest that Brex is a candidate 5-HT7R inverse agonist.

#### 2.1.2. Effects of Brex on Intracellular Signal Transduction Protein in the Plasma Membrane Fraction of Astrocytes

**Figure 3:**
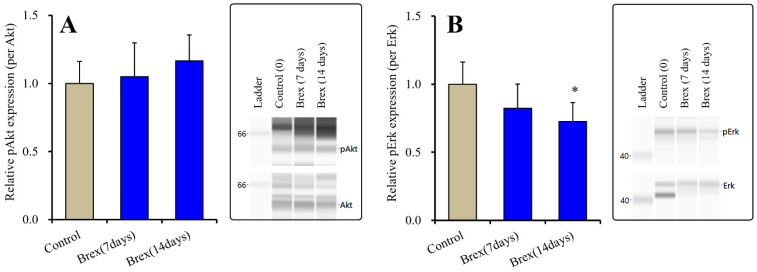
Effects of subchronic administration (7 and 14 days) of therapeutic relevant concentration of Brex (Brex: 300 nM) on protein expression of phosphorylated protein kinase B (pAkt) (panel **A**) and phosphorylated extracellular signal-regulated kinase pErk (panel **B**) in the plasma membrane fraction of cortical primary cultured astrocytes. In left side histograms, ordinate: mean ± SD (*n* = 6) of the relative protein level of pAkt and pErk per GAPDH. * *p* < 0.05: relative to control (Brex-free) by one-way ANOVA with Tukey’s post-hoc test. Right side panels indicate their pseudo-gel images using capillary immunoblotting.

Subchronic administration of a therapeutically relevant concentration of Brex (300 nM) for 7 or 14 days did not affect the protein expression of pAkt (F(2,15) = 1.1(*p* > 0.1)) (Figure 3). Contrary to the results for Akt, Brex (300 nM) administration for 14 days decreased pErk levels, whereas that for 7 days did not affect them (F(2,15) = 4.4(*p* < 0.05)) (Figure 3).

#### 2.1.3. Effects of Brex on pAMPK in the Astroglial Cytosol Fraction

**Figure 4:**
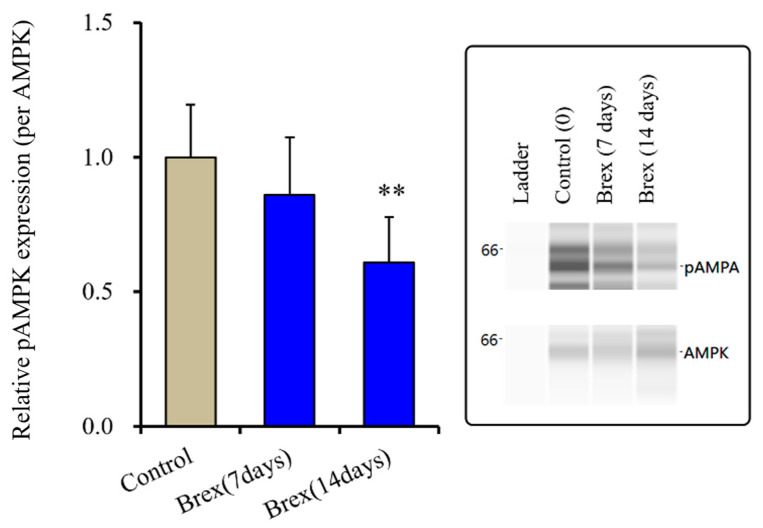
Effects of subchronic administration (7 and 14 days) of therapeutic relevant concentration of Brex (Brex: 300 nM) on protein expression of phosphorylated adenosine monophosphate-activated protein kinase (pAMPK) in the cytosol fraction of cortical primary cultured astrocytes. In left side histograms, ordinate: mean ± SD (*n* = 6) of the relative protein level of pAMPK per GAPDH. ** *p* < 0.01: relative to control (Brex-free) by one-way ANOVA with Tukey’s post-hoc test. Right side panels indicate their pseudo-gel images using capillary immunoblotting.

Subchronic administration of a therapeutically relevant concentration of Brex (300 nM) for 14 days decreased pAMPK levels in the astroglial cytosol fraction, whereas administration for 7 days did not affect them (F(2,15) = 6.4 (*p* < 0.01)) (Figure 4).

### 2.2. Effects of Subchronic Administration of a Therapeutically Relevant Concentration of Brex on Connexin43 Protein Expression in Astrocytes

Subchronic administration of a therapeutically relevant concentration of Brex increased the protein expression of connexin43 in the astroglial plasma membrane [12]. The trafficking process to the plasma membrane of connexin43 is regulated by various protein phosphorylation systems, such as Akt and Erk [14,15,44] signalling [14,15,44]. However, in the present study, subchronic administration of Brex did not affect Akt signalling, while it inhibited Erk signalling. These results suggest that the increased protein expression of connexin43 is probably not modulated by these signalling pathways. Therefore, to clarify the mechanisms behind the increased protein expression of connexin43 in the plasma membrane, the effects of subchronic administration of Brex (300 nM for 14 days) on the protein expression of connexin43 in the astroglial cytosol fraction were examined since the transcription of connexin43 is regulated by AMPK signalling via its activity [34,45].

**Figure 5:**
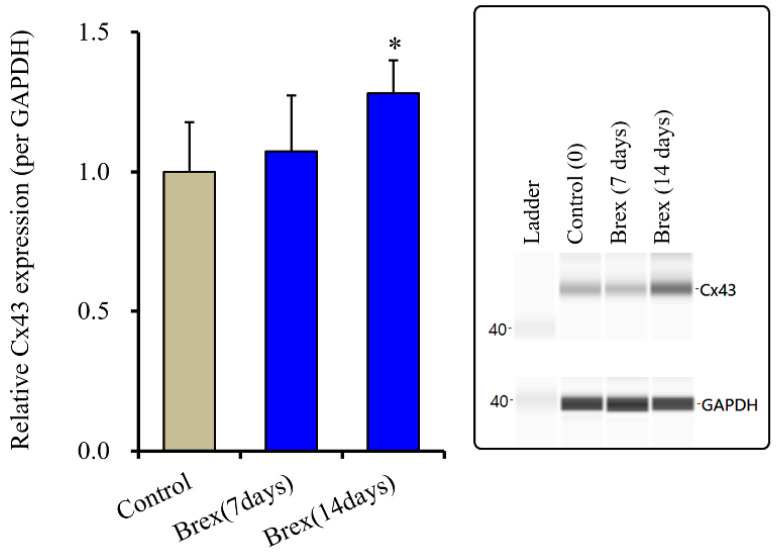
Effects of subchronic administration (7 and 14 days) of therapeutic relevant concentration of Brex (300 nM) on protein expression of connexin43 (Cx43) in the cytosol fraction of cortical primary cultured astrocytes. In left side histograms, ordinate: mean ± SD (*n* = 6) of the relative protein level of Cx43 per GAPDH. * *p* < 0.05: relative to control (Brex-free) by one-way ANOVA with Tukey’s post-hoc test. Right side panels indicate their pseudo-gel images using capillary immunoblotting.

Subchronic administration of Brex (300 nM) for 14 days decreased connexin43 expression in the astroglial cytosol fraction, whereas administration for 7 days did not affect it (F(2,15) = 4.3 (*p* < 0.01)) (Figure 5). 

### 2.3. Effects of Brex on the Intracellular cAMP Level in Astrocytes

Clarifying the specific effects of the downregulation of 5-HT1AR, 5-HT7R, pErk, and pAMPK on intracellular signalling can elucidate the mechanisms underlying various effects of the subchronic administration of Brex. The activation of 5-HT1AR and 5-HT7R decreases and increases cAMP synthesis, respectively [13,46]. Therefore, to clarify the effects of the downregulation of 5-HT1AR and 5-HT7R on downstream intracellular signalling, the concentration-dependent effects of acute administration of a therapeutically relevant concentration of Brex on intracellular cAMP levels in astrocytes were determined.

**Figure 6:**
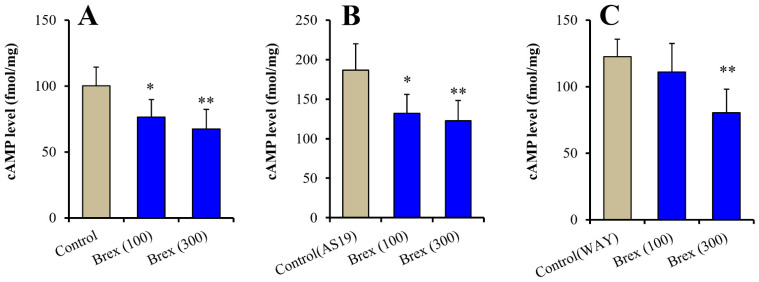
Concentration-dependent acute effects of Brex (100 and 300 nM) on intracellular cAMP level in astrocytes (panel **A**). Interaction among therapeutic relevant concentration of Brex (100 and 300 nM), 5 μM AS19 (5-HT7R agonist) (panel **B**) and 10 μM WAY100635 (5-HT1AR antagonist) (panel **C**) on intracellular cAMP level in astrocytes. Ordinates indicate mean ± SD (*n*  =  6) of intracellular cAMP level in cultured astrocytes (fmol/mg). * *p* < 0.05, ** *p* < 0.01: relative to control (Brex-free) by one-way ANOVA with Tukey’s post-hoc test.

Intracellular cAMP levels in astrocytes were acutely increased by 5 μM AS19 (5-HT7R agonist) but not affected by 10 μM WAY100635 (5-HT1AR antagonist) (F(2,15) = 24.3 (*p* < 0.01)) (Figure 6A (control: Brex-free), Figure 6B (control: AS19 without Brex) and Figure 6C (control: WAY100635 without Brex)). Intracellular cAMP levels in astrocytes were acutely decreased by 100 nM and 300 nM Brex (F(2,15) = 8.6 (*p* < 0.01)) (Figure 6A). On the contrary, both 100 nM and 300 nM Brex suppressed the increased intracellular cAMP level induced by 5 μM AS19 (F(2,15) = 9.1(*p* < 0.01)) (Figure 6B). Under the inhibition of 5-HT1AR by 10 μM WAY100635 (5-HT1AR antagonist), 300 nM Brex decreased the intracellular cAMP level, but 100 nM Brex did not affect it (Figure 6C).

**Figure 7:**
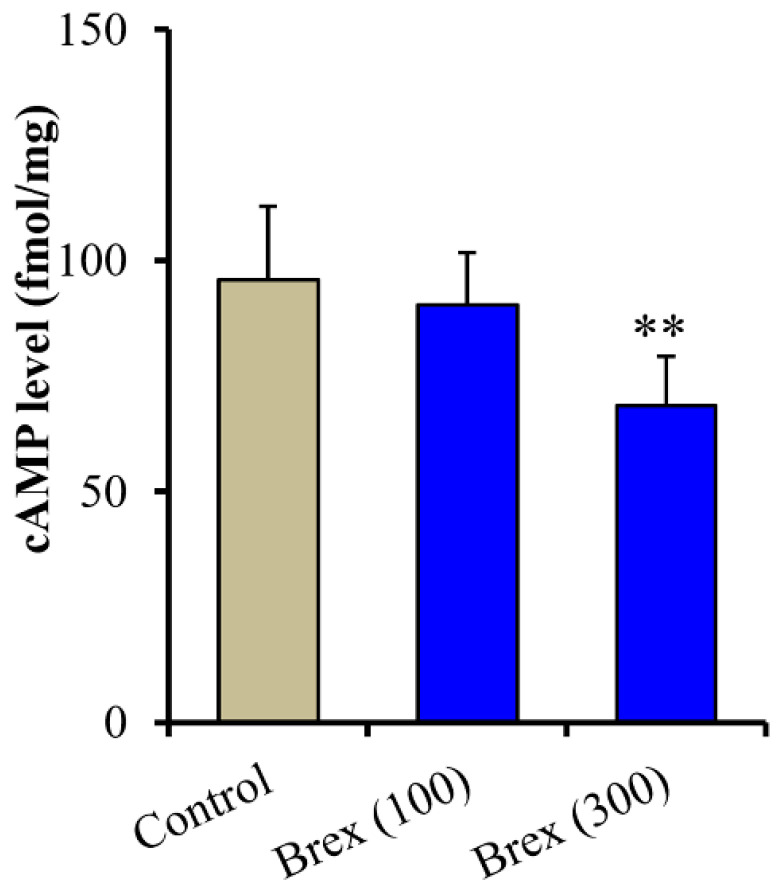
Concentration-dependent subchronic effects of Brex (100 and 300 nM) on intracellular cAMP level in astrocytes. Ordinates indicate mean ± SD (*n*  =  6) of intracellular cAMP level in cultured astrocytes (fmol/mg). ** *p* < 0.01: relative to control (Brex-free) by one-way ANOVA with Tukey’s post-hoc test.

Intracellular cAMP levels in astrocytes were also decreased by the subchronic administration of 100 nM and 300 nM Brex (F(2,15) = 7.7(*p* < 0.01)) (Figure 7).

### 2.4. Effects of Subchronic Systemic Administration of Brex on the cAMP Level and AMPK Signalling in the Hypothalamus In Vivo

#### 2.4.1. Effects of Subchronic Systemic Administration of Brex on pAMPK in the Hypothalamus

**Figure 8:**
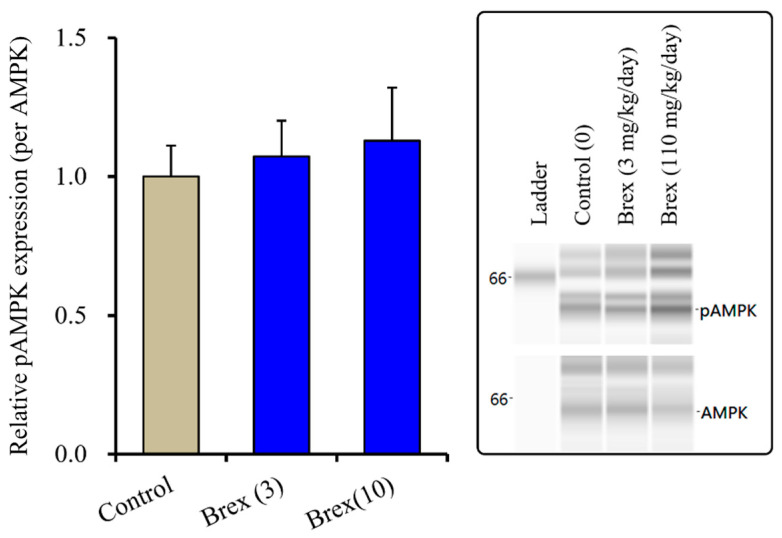
Effects of subchronically systemic administration (14 days) of effective doses of Brex (3 and 10 mg/kg/day) on protein expression of pAMPK in the rat hypothalamus. In left side histograms, ordinate: mean ± SD (*n* = 6) of the relative protein level of pAMPK per GAPDH. Right side panels indicate their pseudo-gel images using capillary immunoblotting.

The suppressive effects of Brex on AMPK signalling could explain the pathophysiology of the low risk of weight gain with Brex [8]. Therefore, to clarify the possible mechanisms of Brex regarding the low risk of weight gain, the effects of subchronic administration of effective doses of Brex on hypothalamic AMPK signalling in vivo were examined. Unexpectedly, subchronic administration of effective doses of Brex (3 and 10 mg/kg/days) for 14 days did not affect the pAMPK level in the rat hypothalamus (Figure 8). 

#### 2.4.2. Effects of Subchronic Systemic Administration of Brex on cAMP Levels in the Hypothalamus

**Figure 9:**
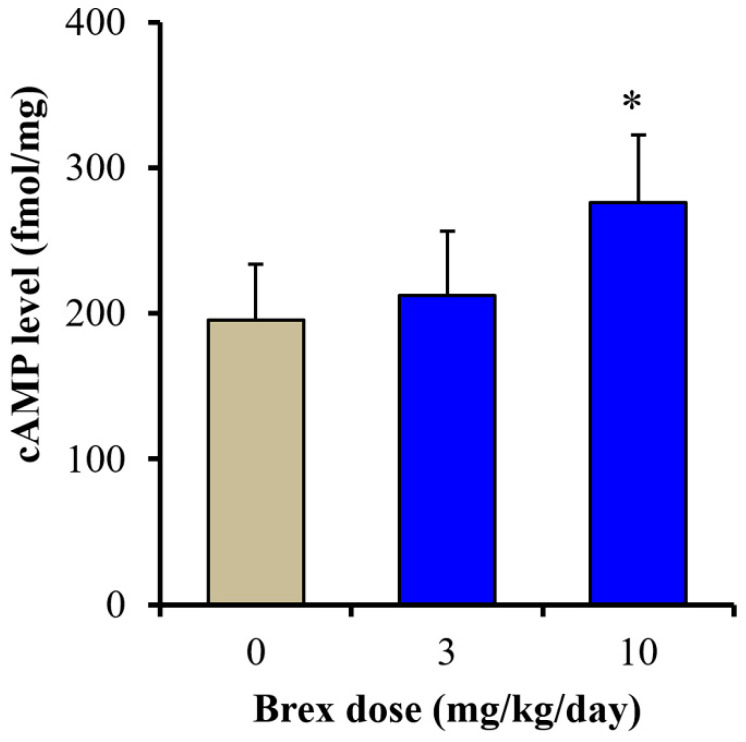
Effects of subchronically systemic administration (14 days) of effective doses of Brex (3 and 10 mg/kg/day) on cAMP levels in the rat hypothalamus. Ordinates indicate mean ± SD (*n*  =  6) of cAMP level in the rat hypothalamus (fmol/mg). * *p* < 0.05: relative to control (Brex-free) by one-way ANOVA with Tukey’s post-hoc test.

To clarify the mechanisms of the discrepant effects of Brex on AMPK signalling, the effects of subchronic administration of effective doses of Brex (3 and 10 mg/kg/days) for 14 days on cAMP synthesis in the rat hypothalamus were examined. Subchronic administration of a therapeutically relevant concentration of Brex (10 mg/kg/day) for 14 days increased the cAMP level in the rat hypothalamus, whereas 3 mg/kg/day of Brex did not affect the cAMP level (Figure 9).

## 3. Discussion

**Figure 10:**
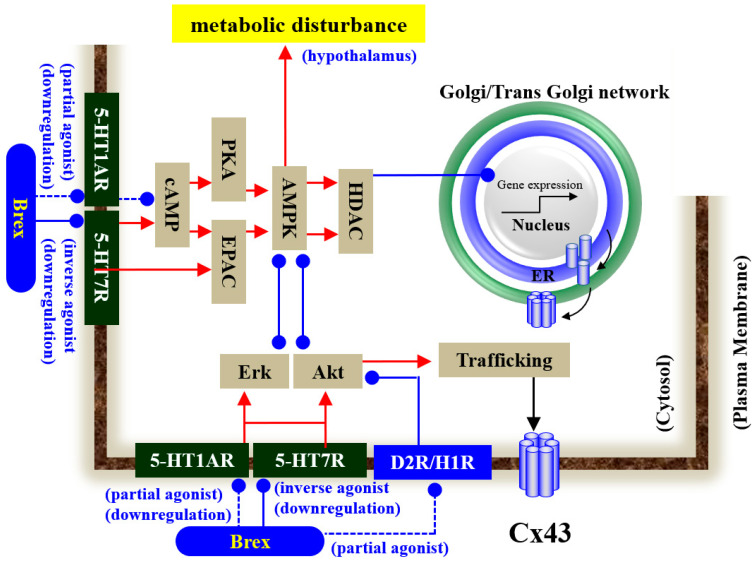
Proposed hypothesis of the mechanisms of Brex on intracellular signalling in astrocytes. Chronic administration of a therapeutically relevant concentration of Brex downregulates both 5-HT1AR and 5-HT7R via its 5-HT1AR partial agonistic and possible 5-HT7R inverse agonistic actions, respectively. The downregulation of 5-HT1AR and 5-HT7R by Brex attenuates AMPK, Erk, and Akt signalling in astrocytes.

Our proposed hypothesis regarding the mechanisms of Brex on astroglial transmission demonstrated by the present and previous studies [12,18,34,41,43] is summarised in Figure 10. demonstrations by in vitro experiments using cultured astrocytes have indicated the possibility that the inhibitory effects of Brex on 5-HT7R play important roles in the clinical actions of Brex via the modulation of astroglial signalling.

### 3.1. Effects of Subchronic Administration of Brex on the Expression of 5-HT1AR and 5-HT7R in Astrocytes

This study demonstrated that the subchronic administration of a therapeutically relevant concentration of Brex affected astroglial transmission through the modulation of 5-HT1AR and 5-HT7R functions. Subchronic administration of a therapeutically relevant concentration of Brex downregulated both astroglial 5-HT1AR and 5-HT7R. These pharmacological features of the subchronic administration of Brex on 5-HT1AR and 5-HT7R seem to be similar to those of lurasidone and vortioxetine [34,43]. It has been revealed that Brex is a 5-HT1AR partial agonist with 60% intrinsic activity [10]. In the present study, acute administration of a therapeutically relevant concentration of Brex decreased the astroglial cAMP level. Activation of 5-HT1AR suppresses cAMP synthesis via the inhibition of adenylate cyclase [12,13,18,34,43,46]. Therefore, these results suggest that the 5-HT1AR partial agonistic action of Brex is at least partly involved in the downregulation of 5-HT1AR. However, in spite of the downregulation of 5-HT1AR, the suppression of cAMP synthesis was continuously observed after the subchronic Brex administration. These results cannot be interpreted as indicating only 5-HT1AR partial agonistic action of Brex.

The mechanisms of downregulation of 5-HT7R induced by subchronic Brex administration require detailed discussion since the binding affinity of Brex to 5-HT7R has been reported, but the function of Brex on 5-HT7R remains to be clarified [10]. Notably, several mood-stabilising atypical antipsychotics, such as olanzapine, clozapine, and lurasidone, are considered to be 5-HT7R inverse agonists since these three mood-stabilising atypical antipsychotics inhibit 5-HT7R but downregulate 5-HT7R expression [34,47]. In the present study, Brex itself acutely decreased the astroglial cAMP level, whereas, under the 5-HT1AR inhibition by WAY100635 (5-HT1AR antagonist), Brex could also decrease the cAMP level. These results suggest that the inhibitory effects of Brex on cAMP synthesis in astrocytes are generated by not only 5-HT1AR partial agonistic action but also some other mechanism. The application of a 5-HT7R agonist, AS19, and an inverse agonist, SB269970, increased and did not affect astroglial cAMP levels, respectively [12,13,18,34,43,46]. Indeed, Brex pretreatment antagonised the AS19-induced increased cAMP level. Therefore, these results suggest that Brex is a 5-HT7R inverse agonist, similar to clozapine, olanzapine, and lurasidone, since Brex inhibits but downregulates 5-HT7R [18,34,47]. Assuming that Brex is a 5-HT7R inverse agonist, the contradictory effect in which subchronic Brex administration continued to suppress cAMP synthesis despite the downregulation of inhibitory 5-HT1AR can be explained by the inhibition and downregulation of excitatory 5-HT7R induced by its inverse agonistic action.

### 3.2. Effects of Subchronic Administration of Brex on Intracellular Signalling in Astrocytes

It is well known that intracellular signalling, such as Erk and Akt signalling, plays important roles in the pathophysiology of mood disorders and cognitive impairments [48] since D2R, 5-HT1A, and 5-HT7R regulate both Akt and Erk [18,49,50,51,52,53] signalling [18,49,50,51,52,53]. Persistent activation of D2R was found to suppress Akt signalling via the dephosphorylation of Akt [52], and Akt-deficit mice displayed impairment of prepulse inhibition [54]. In contrast, a selective 5-HT transporter inhibitor enhanced Akt phosphorylation via the activation of 5-HT1AR [55]. Furthermore, subchronic applications of haloperidol and mood-stabilising atypical antipsychotics, such as clozapine, olanzapine, quetiapine, risperidone, and zotepine, activate Akt signalling [12,41,52,53,54]. In contrast to Akt signalling, acute applications of haloperidol, clozapine, and risperidone acutely activate Erk signalling in hippocampal neurons [50,51], whereas these effects were not observed in D2R-deficit mice [49]. The activation of 5-HT1AR also enhances the phosphorylation of Erk [56].

Based on these previous findings and the binding profile of Brex, a high-affinity partial agonist to D2R and 5-HT1AR, we speculated that Brex increases both Erk and Akt signalling. However, contrary to our expectations, subchronic administration of a therapeutically relevant concentration of Brex did not enhance Akt or Erk signalling. These contradictions between the previous findings and the present results in interpreting the pathophysiology of Brex suggest that a 5-HT7R-inverse agonist-like action of Brex, with inhibition of 5-HT7R function and downregulation of 5-HT7R expression, probably provides the novel pathophysiological strategies [13]. The basis of our hypothesis regarding 5-HT7R inverse agonists has already been supported by lurasidone and vortioxetine [18,34,43,57,58,59]. Subchronic administration of a therapeutically relevant concentration of lurasidone and vortioxetine suppressed Erk signalling without affecting Akt signalling [18,34,43]. Similar to these two agents, a selective 5-HT7R inverse agonist, SB269970, also inhibited Akt and Erk [18,60] signalling [18,60]. In particular, SB269970 rapidly downregulated 5-HT1AR, 5-HT7R, and Erk signalling [18,43] and generated rapid-acting anti-immobility-like and antidepressant-like effects [61]. Taken together with these previous findings, the present results suggest that the activation of Akt and Erk signalling is not necessarily a mechanism of the clinical action of mood-stabilising atypical antipsychotics. Rather, recent preclinical studies suggest that hyperactivation of Akt signalling plays an important role in the pathophysiology of several serious adverse reactions induced by clozapine, such as cardiotoxicity, non-infectious pneumonia, convulsions, and metabolic syndrome [12,19,28,44].

### 3.3. Impacts of the Suppression of AMPK Signalling on Tripartite Synaptic Transmission

In our previous study, several mood-stabilising atypical antipsychotics, such as clozapine, quetiapine, and zotepine, enhanced the trafficking of connexin43 to the plasma membrane via the activation of Akt signalling [12,28,40]; exceptionally, Brex weakly increased protein expression of connexin43 in the astroglial plasma membrane, but this trafficking process was independent of Akt signalling [12]. 

On the contrary, vortioxetine and lurasidone enhanced connexin43 expression in astroglial cytosol via the inhibition of AMPK signalling [18,34]. It has been established that AMPK signalling regulates the transcription of various ion channels via the activation of histone deacetylase [45]. The transcription of connexin43 is also regulated by AMPK signalling [34,62]. To clarify the mechanisms by which Brex induced increased connexin43 expression in the astroglial plasma membrane, the present study examined the effects of subchronic administration of Brex on astroglial AMPK signalling and protein expression of connexin43 in the cytosol fraction. In accordance with our expectations, Brex increased the connexin43 level in the cytosol fraction and suppressed AMPK signalling. These actions of Brex on connexin43 are similar to those of the histone deacetylase inhibitor valproate [12,28].

The clinical features of Brex for the treatment of mood disorders involve dominant antidepressant-like action compared to other antipsychotics [4,5,6,7]. Mood-stabilising antipsychotics (clozapine, quetiapine, and zotepine) enhance astroglial L-glutamate release through activated connexin43-containing hemichannels [12,28,41], whereas 5-HT transporter-inhibiting antidepressants and lurasidone suppress astroglial L-glutamate release via the suppression of astroglial connexin43-containing hemichannel activities [13,15,18,43,44]. Therefore, the enhancement and suppression of connexin43 function probably contribute to mood-stabilising and antidepressive actions, respectively [15,18,34,44]. Considering the previous findings, the antidepressive-dominant mood-stabilising feature of Brex is probably, at least partially, involved in the modulation of tripartite synaptic transmission via 5-HT7R and AMPK signalling.

### 3.4. Clinical Implication and Low Risk of Metabolic Syndrome 

A recent meta-analysis study reported that Brex is among the better antipsychotics associated with a low risk of metabolic outcomes [8]. AMPK signalling activator in peripheral organs is considered to be a promising candidate anti-obesity agent due to its activation stimulating muscle and hepatic mitochondrial biogenesis and fatty acid oxidation [63]; however, contrary to peripheral organs, in the central nervous system, activation of AMPK signalling in the hypothalamus enhances the secretion of glucagon, corticosterone, and epinephrine, resulting in the activation of hepatic gluconeogenesis and glycogenolysis [64]. AMPK activity in the hypothalamus is regulated by nutrients, anorexigenic and orexigenic signalling, cAMP/PKA, and histamine H1 receptor (H1R) [65,66]. The hypothalamus, which is the main sensor of nutrient concentrations, receives afferents (dopamine, norepinephrine, 5-HT, and histamine) from various basal brain regions [65]. 

Initially, the weight gain risks of Brex were speculated [67] since the affinity of Brex to H1R was Ki = 19 nM [10] (Table 1). The inhibition of H1R is considered to be involved in the high risk of weight gain of several atypical antipsychotics via activation of AMPK signalling [65]. Indeed, both clozapine and olanzapine (high-affinity H1R antagonists [65,66]) induced weight gain was compensated by H1R agonist via suppression of AMPK signalling [68,69,70], whereas lurasidone (low binding affinity to H1R) suppresses AMPK signalling in the hypothalamus [34]. Although we could not identify the expression of histamine H1R in the astroglial plasma membrane, therefore, the suppressive effects of Brex on astroglial AMPK signalling cannot deny being over-evaluated due to the lack of expression of H1R in astrocytes. Therefore, to clarify the actual mechanisms of low risk of weight gain of Brex, the subchronic administration of effective dose of Brex (10 mg/kg/day) for 14 days on cAMP levels and AMPK signalling in rat hypothalamus were determined [10,71]. The lack of activation of AMPK signalling in astrocytes and hypothalamus can explain, at least partially, the mechanisms of the low risk of weight gain property of Brex; however, the discrepant effects of Brex on AMPK signalling between astrocyte and hypothalamus need some discussion.

The D2R (Ki = 0.3 nM) and 5-HT1AR (Ki = 0.12 nM) partial agonistic actions of Brex contribute to increasing cAMP level, whereas 5-HT7R inverse agonist-like action (Ki = 3.7 nM) decreases cAMP level (Table 1). Considering these receptor binding profiles of Brex, it is probably a reasonable demonstration that Brex decreased cAMP levels in the astrocytes. Although the results demonstrated by in vitro astroglial experiments strongly suggested that the 5-HT7R inverse agonist-like action of Brex was a candidate mechanism, similar to lurasidone, the results of in vivo hypothalamus experiments deny our expectations since subchronically systemic administration of Brex increased cAMP levels in the hypothalamus. The direct interaction between 5-HT7R and H1R on intracellular signalling has remained to be clarified since the major second messenger system of H1R is inositol phosphate [72,73]. However, activation of H1R weakly increases cAMP synthesis via G_βγ_ subunits from G proteins [73]. Therefore, the binding profile of Brex to these four receptors (D2R, 5-HT1AR, 5-HT7R, and H1R) alone cannot fully explain the effects of Brex on AMPK signalling or cAMP synthesis in the hypothalamus. Additionally, both Akt and Erk signallings also contribute poorly to the effects of Brex on AMPK signalling since these signallings form a negative feedback loop with AMPK signalling [74,75,76].

The present study demonstrated that the effects of Brex on tripartite synaptic transmission are probably y generated by complicated mechanisms compared to those of clozapine, quetiapine, zotepine, and lurasidone [12,28,41]. The therapeutic relevant concentration of Brex weakly enhanced astroglial L-glutamate release through connexin43-containing hemichannel, similar to clozapine, quetiapine, and zotepine [12,28,41]; however, the effect of Brex on trafficking and transcription processes of connexin43 was rather similar to lurasidone and vortioxetine, which suppress connexin43 function via their 5-HT7R inverse agonistic action [18,34]. These results suggest that the 5-HT7R inverse agonist-like action of Brex cannot be ignored as a mechanism of antidepressive-dominant mood-stabilising effects of Brex. In contrast to mechanisms of mood-stabilising action, the lack of stimulatory effects on AMPK signalling (decreased and did not affect AMPK signalling in respective astrocyte and hypothalamus) possibly contributes to a part of the mechanism of low risk of weight gain of Brex; however, rather, the results in the present study strongly indicate the existence of another major mechanism. Therefore, exploring the effects of Brex on the AMPK signalling cascade can identify the novel mechanisms of mood-stabilising action and low risk of weight gain of Brex. We shall explore the effects of acute and chronic effects of Brex on AMPK signalling cascade using in vivo study in the future.

## 4. Materials and Methods

### 4.1. Chemical Agents and Drug Administration

Brex was obtained from Funakoshi (Tokyo, Japan). 5-HT1AR antagonist, WAY100635, 5-HT7R agonist, and AS19 [77] were obtained from CosmoBio (Tokyo, Japan). These three agents were prepared on the day of the experiment and were initially dissolved in dimethyl sulfoxide at 25 mM. The final dimethyl sulfoxide concentration was lower than 0.1% (vol/vol).

The therapeutic relevant serum concentration of Brex was reported to range from 90 nM to 300 nM [35,36]. Based on the clinical findings, in the present study, cultured astrocytes were administrated by 300 nM Brex for 7 or 14 days [12]. According to previous studies, the cultured astrocytes were administrated by 10 μM WAY100635 and 5 μM AS19. Previous studies have reported that the effective dose of Brex was 3 or 10 mg/kg/day [10,71]. Based on the previous reports, in the present study, to explore the dose-dependent effects of systemic subchronic administration of Brex on cAMP levels and AMPK signalling in the rat hypothalamus, rats were subcutaneously administered Brex (3 or 10 mg/kg/day) for 7 and 14 days using an osmotic pump (2ML_1 and 2ML_2, Alzet, Cupertino, CA, USA).

### 4.2. Preparation of Primary Astrocyte Culture

All animal care and experimental procedures described in this report were performed according to the ethical guidelines established by the Institutional Animal Care and Use Committee at Mie University, Japan (no. 2019-3, 24 May 2019) and are reported in accordance with the Animal Research: Reporting of In vivo Experiments (ARRIVE) guidelines [78]. Mainly, the preparation of astrocytes followed the protocol of the previous study [12,18,34,43]. Pregnant Sprague–Dawley rats (SLC, Shizuoka, Japan) were housed individually in cages, which were kept in air-conditioned rooms (temperature, 22 ± 2 °C) with a 12 h light/dark cycle and free access to food and water. Cultured astrocytes were prepared from cortical astrocyte cultures of neonatal Sprague–Dawley rats (*n* = 36), which were sacrificed by decapitation at 0–48 hr of age. The cerebral hemispheres were removed using a dissecting microscope. The cerebral tissue was chopped into fine pieces using scissors and then triturated briefly with a micropipette. The suspension was filtered using 70 µm nylon mesh (BD, Franklin Lakes, NJ, USA) and centrifuged. Then, the pellets were resuspended in 10 mL Dulbecco’s modified Eagle’s medium (D6546; Sigma-Aldrich, St. Louis, MO, USA) containing 10% foetal calf serum (fDMEM); this procedure was repeated three times. The day after culturing for 14 days (DIV14), contaminated cells were removed by shaking in a standard incubator for 16 h at 200 rpm. Astrocytes were removed from flasks by trypsinisation and seeded directly onto a translucent polyethylene terephthalate (PET) membrane (1.0 μm) with 24 well plates (BD) at a density of 100 cells cm^2^ for experiments from DIV14 to DIV28, the culture medium (fDMEM) was changed twice a week, and Brex (300 nM) was administered subchronically (for 7 days, DIV21–28; or for 14 days, DIV14–28) [79]. Previously, our methods detected that the remaining adherent cells included more than 90% glial fibrillary acidic protein-positive and A2B5-negative cells, as detected using immunohistochemical staining [39].

To determine the acute effects of WAY100635, AS19, and Brex on intracellular cAMP levels on DIV28, after the washout, cultured astrocytes, which were incubated in fDMEM without any Brex, were incubated in ACSF containing 10 μM WAY100635 (selective 5-HT1AR antagonist), 5 μM AS19 (selective 5-HT7R agonist) plus 100 nM or 300 nM Brex for 120 min (acute administration) [12,18,34].

### 4.3. Extraction of Cultured Astrocytes and Rat Hypothalamus

On DIV28, the cultured astrocytes were washed out using ACSF (comprised NaCl 150.0 mM, KCl 3.0 mM, CaCl_2_ 1.4 mM, MgCl_2_ 0.8 mM, and glucose 5.5 mM and buffered to pH 7.3 with 20 mM HEPES buffer), and this procedure was repeated three times [79]. After the subchronic administration of effective doses of Brex [43,58,59], the rat hypothalamus was dissected according to the method described by Glowinski and Iversen [80]. To apply the capillary immunoblotting system, after the washout, the cytosol and plasma membrane fractions of cultured astrocytes and dissected rat hypothalamus were extracted using a Minute Plasma Membrane Protein Isolation Kit (Invent Biotechnologies, Plymouth, MN, USA). To determine the intracellular cAMP level, after the washout, the cultured astrocytes and dissected rat hypothalamus were placed into respective 0.5 mL and 1.5 mL microtubes and homogenised with an ultrasonic cell disrupter (VP-050N, Taitec, Koshigaya, Japan) in chilled 0.1 N HCl. The mixture was centrifuged at 10,000× *g* for 20 min at 4 °C. Filtered aliquots (5 μL) were injected into the ultra-high-performance liquid chromatography (UHPLC) with a mass spectrometry system (LCMS).

### 4.4. Capillary Immunoblotting Analysis

Capillary immunoblotting analysis was performed using Wes (ProteinSimple, Santa Clara, CA, USA), according to the manufacturer’s instructions. The lysates of the primary cultured astrocytes and hypothalamus were mixed with a master mix (ProteinSimple) to obtain a final concentration of 1× sample buffer, 1× fluorescent molecular weight marker, and 40 mM dithiothreitol; the mixture was heated at 95 °C for 5 min. The samples, blocking reagents, primary antibodies, HRP-conjugated secondary antibodies, chemiluminescent substrate (SuperSignal West Femto; Thermo Fisher Scientific, Waltham, MA, USA), and separation and stacking matrices were also dispensed into the designated wells of a 25-well plate. After plate loading, separation electrophoresis and immunodetection steps were performed in the capillary system, which was fully automated. Capillary immunoblotting analysis was conducted at room temperature, and the instrument’s default settings were used. Capillaries were first filled with a separation matrix, followed by a stacking matrix, with approximately 40 nL of the sample used for loading. During electrophoresis, the proteins were separated by molecular weight through the stacking and separation matrices at 250 V for 40–50 min and then immobilised on the capillary wall using proprietary photo-activated capture chemistry. Then, the matrices were washed out. Next, the capillaries were incubated with a blocking reagent for 15 min, and the target proteins were probed with primary antibodies, followed by HRP-conjugated secondary antibodies (Anti-Rabbit IgG HRP, A00098, 10 μg/mL, GenScript, Piscataway, NJ, USA). Antibodies against GAPDH (NB300-327, 1:300, Novus Biologicals, Littleton, CO, USA), connexin43 (C6219, 1:100, Sigma-Aldrich, St. Louis, MO, USA), 5-HT1AR (NBP2-21590, 1:00, Novus Biologicals, Littleton, CO, USA), 5-HT7R (NB100-56352, 1:00, Novus Biologicals, Littleton, CO, USA), Erk (AF1576, 10 μg/mL, R&D systems, Minneapolis, MN, USA), phosphorylated Erk (AF1018, 5 μg/mL, R&D Systems), Akt (AF1775, 1 μg/mL, R&D Systems), phosphorylated Akt (AF877, 5 μg/mL, R&D Systems), AMPKα (2603, 1:50, Cell Signalling Technology, Danvers, MA, USA), and phosphorylated-AMPKα (2535, 1:50, Cell Signalling Technology, Danvers, MA, USA) were diluted in an antibody diluent (Immuno Shot Platinum, CosmoBio, Tokyo, Japan) [79].

### 4.5. Determination of Intracellular cAMP Levels in Cultured Astrocytes and Rat Hypothalamus 

The cAMP levels were determined by UHPLC (Acquity UPLC H-Class system; Waters, Milford, MA, USA) with mass spectrometry (Acquity SQ detector; Waters, Milford, MA, USA). Five microlitres of filtered samples was injected using an autosampler (Acquity UPLC Sample Manager FTN; Waters, Milford, MA, USA). cAMP was separated by UHPLC equipped with a graphite carbon column (particle 3 μm, 150 × 2.1 mm; Hypercarb, Thermo, Waltham, MA, USA) at 40 °C, and the mobile phase was set at 450 µL/min [40]. A linear gradient elution programme was used for over 10 min with mobile phases A (1 mM ammonium acetate buffer, pH 11) and B (acetonitrile). The nitrogen flows of the desolvation and cone were set at 750 and 5 L/h, respectively, and the desolvation temperature was set at 450 °C. The cone voltage for the determination of cAMP (*m/z* = 330.3) was 42 V.

### 4.6. Data Analysis

All experiments in this study were designed with equally sized animal groups (*n* = 6) without conducting a formal power analysis, in accordance with previous studies [18,43,58,59]. All values are expressed as the mean ± standard deviation (SD), and a *p*-value < 0.05 (two-tailed) was considered statistically significant for all tests. Drug levels for acute, subchronic, and chronic administrations were selected on the basis of values reported in previous studies. Where possible, we aimed to randomise and blind the data. Particularly, for the determination of cAMP levels and protein expression, the sample order on the autosampler and Wes was determined using a random number table. 

Time-dependent effects of subchronic and chronic administrations of Brex on protein levels (connexin43, 5-HT1AR, 5-HT7R, phosphorylated ERK, and phosphorylated AKT) in the cytosol and plasma membrane fractions were analysed by one-way analysis of variance (ANOVA) with Tukey’s multiple comparison, using Bell Curve for Excel ver3.2 (Social Survey Research Information Co., Ltd., Tokyo, Japan). The interaction between Brex and WAY100635 on protein expression was analysed by Student’s *T*-test using Bell Curve for Excel. The concentration-dependent and dose-dependent effects of Brex on cAMP levels and pAMPK levels were also analysed using one-way ANOVA with Tukey’s multiple comparison. The data and statistical analysis comply with the recommendations of the British Journal of Pharmacology on experimental design and analysis in pharmacology [81].

### 4.7. Nomenclature of Targets and Ligands

Key protein targets and ligands in this report are hyperlinked to corresponding entries in http://www.guidetopharmacology.org, (accessed on 1 June 2020) common portal for data from the IUPHAR/BPS Guide to PHARMACOLOGY (Harding et al., 2018), and are permanently archived in the Concise Guide to PHARMACOLOGY 202/22 [10,34,65,72,74,75,77].

## 5. Conclusions

The present study determined the effects of subchronic administration of therapeutic relevant concentration of Brex on astroglial signalling-associated 5-HT receptors to explore mechanisms underlying mood-stabilising antipsychotic effects and low risk of weight gain by Brex. Subchronic administrations of therapeutic relevant concentrations of Brex downregulated both 5-HT1A and 5-HT7R. The response of downregulation of 5-HT7R was faster than that of 5-HT1AR since the downregulations of 5-HT1A and 5-HT7R were observed for 14 days and 7 days, respectively. In the low range of therapeutic relevant concentration of Brex (100 nM), acutely, the 5-HT1AR partial agonistic action was predominant, but in the high range, the 5-HT7R inverse agonistic action was added to the 5-HT1A partial agonistic action. These concentration-dependent complicated actions of Brex contribute to various intracellular signallings in astrocytes. The intracellular signalling of Erk and AMPK were also downregulated by subchronic administration of therapeutic relevant concentration of Brex for 14 days (synchronised with the downregulation of 5-HT1AR), whereas Akt signalling was not affected by Brex. Contrary to in vitro studies, systemically subchronic administration of effective doses of Brex did not affect AMPK signalling but increased cAMP levels in the hypothalamus. These discrepant results between in vitro (astrocyte) and in vivo (thalamus) suggest that the D2R partial agonistic with low intrinsic activity and H1R antagonistic actions of Brex, which lead to weight gain via activation of thalamic AMPK signallings, is probably suppressed by other mechanisms, such as 5-HT7R inverse agonist-like action.

## Figures and Tables

**Table 1:** Receptor binding profiles of mood-stabilising atypical antipsychotics. Brexpiprazole (Brex), aripiprazole (APZ), clozapine (CLZ), lurasidone (LUR), quetiapine (QTP), ziprasidone (ZIP), zotepine (ZTP) against serotonin (5-HT) type 1A (5-HT1AR), type 2A (5-HT2AR), type 7 (5-HT7R) receptor, histamine type 1 (H1R) receptor and dopamine receptors type 1 (D1R) and 2 (D2R). Data are equilibrium constant (Ki) values (nM).

Receptor	Brex	APZ	CLZ	LUR	QTP	ZIP	ZTP
5-HT1AR	0.12	5.6	124	6.8	432	2.5	471
5-HT2AR	0.47	8.7	5.4	2.0	100	0.08	2.7
5-HT7R	3.7	10.3	18.0	0.5	307	6	12.0
H1R	19	27.9	1.13	>1000	2.2–11	15	3.21
D1R	160	>1000	266	262	712	30	71.0
D2R	0.3	3.3	157	1.7	245	4.6	25.0
Reference	[10]	[20,21]	[22,23]	[24]	[25]	[26]	[27]

## Data Availability

The data that support the findings of this study are available from the corresponding author upon reasonable request. Some data may not be made available because of ethical restrictions.
